# Resilience and Wellbeing in Mental Health Workforce: Why it Matters and How to Develop it

**DOI:** 10.1192/j.eurpsy.2022.48

**Published:** 2022-09-01

**Authors:** C. Steinebach

**Affiliations:** Zurich University of Applied Sciences, School of Applied Psychology, Zürich, Switzerland

## Abstract

Resilience, mental health, and well-being are currently being discussed in general and for healthcare workers. Employees in the mental health workforce are subject to a variety of stresses. There have been long-standing stresses due to structural and discipline-specific conditions. In addition, there are special challenges due to the pandemic.

The systemic perspective of the biopsychosocial model of human development focuses on biochemical, muscular-neuronal, emotional, cognitive, and environmental risk and protective factors. These systemic events are embedded in different environmental systems that represent micro- to macrosystemic conditions. All these factors need to be reflected, evaluated, and positively developed in a profession- and workplace-specific manner. In this sense, “career resilience” can be promoted individually, at team level and organizationally. Corresponding evidence-based programs for prevention and intervention are presented. In the discussion, it becomes clear that expectations of societal requirements can also be derived under the concern of prevention and promotion. It is necessary to complement self-care with the “caring team community” to promote the development of a comprehensive “caring society”. Thus, we contribute to an overarching conception in terms of transdisciplinary consilience of resilience and wellbeing in mental health workforce.

**Disclosure:**

No significant relationships.

